# Postural Instability in Parkinson’s Disease: A Review

**DOI:** 10.3390/brainsci9090239

**Published:** 2019-09-18

**Authors:** Bhavana Palakurthi, Sindhu Preetham Burugupally

**Affiliations:** 1 Department of Biological Sciences, University of Notre Dame, Notre Dame, IN 46556, USA; bpalakur@nd.edu; 2 Department of Mechanical Engineering, Wichita State University, Wichita, KS 67260, USA

**Keywords:** postural instability (PI), Parkinson’s disease (PD), PD factors, PD diagnosis

## Abstract

Parkinson’s disease (PD) is a heterogeneous progressive neurodegenerative disorder, which typically affects older adults; it is predicted that by 2030 about 3% of the world population above 65 years of age is likely to be affected. At present, the diagnosis of PD is clinical, subjective, nonspecific, and often inadequate. There is a need to quantify the PD factors for an objective disease assessment. Among the various factors, postural instability (PI) is unresponsive to the existing treatment strategies resulting in morbidity. In this work, we review the physiology and pathophysiology of postural balance that is essential to treat PI among PD patients. Specifically, we discuss some of the reported factors for an early PI diagnosis, including age, nervous system lesions, genetic mutations, abnormal proprioception, impaired reflexes, and altered biomechanics. Though the contributing factors to PI have been identified, how their quantification to grade PI severity in a patient can help in treatment is not fully understood. By contextualizing the contributing factors, we aim to assist the future research efforts that underpin posturographical and histopathological studies to measure PI in PD. Once the pathology of PI is established, effective diagnostic tools and treatment strategies could be developed to curtail patient falls.

## Highlights

- PI diagnosis remains a complex clinical task, and PI treatment is often unresponsive to the existing treatments such as drugs, surgery, and physiotherapy.
- Screening for Vitamin D deficiency and LRRK2 mutations might help estimate PI risk in PD patients.
- Measuring center of gravity (CoG), center of pressure (CoP), fear of fall (FoF), and abnormal proprioception might help grade PI severity.
- Imaging white matter hyperintensities (WMHs), Gray Matter (GM) atrophy, and basal ganglion abnormalities remain the best available diagnostic tests for identifying pathological lesions contributing to PI.

## 1. Introduction

The geriatric population is commonly afflicted with gait and balance disorders such as Parkinson’s disease (PD). PD is a progressive neurodegenerative movement disorder caused by the degeneration of substantia nigra dopaminergic neurons present in basal ganglia. The symptoms of PD are predominantly motor-based such as tremor, rigidity, bradykinesia, postural instability, hypomimia, micrographia, festination, shuffling gait, dysarthria, and dystonia. The nonmotor symptoms are autonomic dysfunction, cognitive abnormalities, dementia, sleep disorders, anosmia, and pain [1,2,3].

PD patients differ in their clinical signs, natural history, genetic makeup, age at onset, rate of disease progression, and response to treatment [2,4,5,6], making PD a heterogeneous disorder. Such heterogeneity has been used to categorize PD patients. Foltynie et al. explored the concept of heterogeneity in PD and observed that subtype classifications were based on clinical signs at presentation, genotypes, the rate of progression, and the age of onset [7]. Of all the classifications, subtype classification based on phenotypes has been accepted widely [8]. According to phenotypic categorization, tremor dominant (TD), postural instability and gait difficulty (PIGD) or akinetic rigid (AR), and mixed [5,9,10] are the major subtypes. This phenotypic classification is based off a validated hypothesis: the ratio of the tremor item score and the PI and gait difficulty items’ score calculated from the Unified Parkinson Disease Rating Scale (UPDRS) criteria [5,11,12,13]. Thus, it is evident that PI is one of the most important criteria for diagnosing and categorizing PD patients. 

Earlier PI has been identified as a feature of late-stage PD, labeling the onset of moderate to severe stages, as scaled on the Hoehn & Yahr criterion, at stages 3 and above [4]. But now, it is regarded as a presenting symptom of the PIGD subtype, frequently associated with gait difficulty and falls [5]. PI in PD is the inability to balance due to loss of postural reflexes [2]—specifically, balance reaction, adoption of a flexed posture, and trunk rotation [6]. These motor impairments are caused by dopaminergic neuronal deficits, comorbid white matter disease, and cholinergic system degeneration [14]. 

PI among PD patients is unresponsive to all kinds of treatments available [15]. Instability often leads to falls, the most common reason for emergency room visits and the largest motor related contributor to health care costs in PD patients [16]. Early identification of instability, persistent monitoring, and effective timely intervention is necessary to curb the growing economical and emotional burden of PI in PD patients. The increase in falls reflects the diagnostic inadequacy, which in turn is due to inadequate characterization and quantification of the pathogenic factors [16].

Characterization refers to the identification of factors that cause PI, which is a multifactorial disorder [17]. Targeting patient-specific factors is very important for an effective treatment. Currently, the diagnosis is subjective. Though the objective nature of assessment will result in a more efficient diagnosis, it is an expensive and a time-consuming process [16]. Hence, there is a need to develop cost effective and foolproof diagnostic tools for measuring PI. 

Here, we review the factors that have been identified to cause PI in PD patients, which might help to bridge the gap in optimizing a diagnostic tool for PI. We also briefly discuss the epidemiological facts and the advancements in the management of PI.

## 2. Epidemiology

Epidemiological facts reflect the functional and economical concerns of PIPD on the society. PD is the most common neurodegenerative disease after Alzheimer’s with an annual incidence of 15 per 100,000 [18]. Its prevalence in the general population is 0.3% [19], and it is 1% in those above 65 years [18]. Nearly 20% of patients report a family history of PD [20], and the gene–environmental interactions increase its odds ratio from 1.6 to 12.6 for combinations like smoking and α-synuclein or coffee and apolipoprotein E [12]. 

PI occurs in 16% of the patients [21,22]. Seventy-seven percent of pathologically proven PD patients initially respond to dopamine replacement therapy, with PI being refractory to the treatment [2,15]. As the disease progresses, postural instability worsens and often leads to falls [15,23]. Falls occur in nearly 60% of PD patients [24], and about 75% of the total hospitalizations in PD patients internationally are attributed to falls or fractures [25].

## 3. Physiology of Postural Balance Control

Postural balance control is a multifactorial, nonvolitional activity. Postural stability is the ability to maintain equilibrium under both static and dynamic conditions such as during quiet stance, perturbations, and preparation of movements [26]. Adequate sensory and motor system coordination to prepare, adjust (perception of body schema), and execute a movement is needed to maintain postural stability [27,28]. Perception depends on a multimodal sensory input to the CNS about the position of each joint with respect to other parts of the body [29]. Stabilization of posture control is a closed-loop circuit formed by integration of the brain stem, spinal cord networks, and cognitive and sensory feedback [14,30,31,32,33,34]. Of note, basal ganglia through direct and indirect pathways facilitate stable execution of agonist (activation) and antagonist (inhibition) muscles [35,36].

Hence, it is very clear that postural stability is dependent on the integration of sensory, motor, visual, vestibular, and cognitive circuits, and disruption in any of them leads to PI. Learning the physiology of balance control will help rightly identify derailed factors from signs elicited by a patient with loss of postural balance.

## 4. Pathophysiology

### 4.1. Pathogenesis

Learning physiology of postural balance has made it clear that the basal ganglion is a critical part of maintaining balance. As basal ganglia are hypodopaminergic in PD, there is PI. However, some PI patients remain unresponsive to dopamine replacement therapies [37] supporting nondopaminergic involvement. The theory of hypodopaminergic pathology has been extended to a multisystem neurodegeneration, cortical amyloid deposition [31], differential disruption of the corpus callosal fibers [38], and periventricular white matter hyperintensities [39] to name a few.

With defective basal ganglion related balance maintenance, PD patients need a greater compensatory input from other parts of the cognitive/sensory/motor regions of the brain for better stability and orientation [40]. For instance, posturographic studies have shown that severely affected PD patients depend on their vision for maintaining postural stability [41]. Any reduced peripheral sensation, vision disturbance, labyrinthine dysfunction, and lack of predictability to environmental perturbance exacerbates PI in PD patients [17,28,42]. PI progression is faster in TD and mixed subtypes in late onset cases [43] and in the G2019S mutation carrying early onset PD cases [44]. 

### 4.2. Diagnosis 

Diagnosis of PI is made clinically by a neurologist or a movement disorder specialist and is purely subjective. There have been multiple clinical tests, assessment scales, bio and biomechanical markers, and gait predictability techniques developed to diagnose and scale PI.

Clinical tests such as tandem Romberg stance and balancing on one foot, pull tests, push–pull tests, timed up and go tests, functional reach tests, and Tinetti and Simon tasks are in use [45,46]. Instruments such as force plates, stabilograms, clinical posturography, foot switches, GaitRite mats, and wearable sensors are routinely used to perform these clinical tests [46]. The quantitative tests used routinely are kinematic gait analysis, global mobility task, phase coordination index, ambulatory gait assessment, ambulatory freezing assessment, and the GAITRite system [47]. The pull test (PT), is reported to have the highest specificity and sensitivity when rightly performed and interpreted [48]. However, many studies have concluded that multiple balance tests assessing the different types of postural stress provide an optimal assessment of PI [48,49].

Some of the scales used for assessment are Tinetti balance, Berg balance scale, UPDRS, activities of balance confidence, Schwab and England scale, and the balance evaluation system test (BEST) [45]. The Unified Parkinson’s Disease Rating Scale (UPDRS) is used to score the clinical severity of disease [49]. The activities-specific balance confidence (ABC) scale uses a questionnaire to score the confidence on balance control and estimate the fear of fall [50]. The Schwab and England test also uses a questionnaire to evaluate the patient’s ability to perform activities of daily living in terms of speed and independence [51].

Functional magnetic resonance imaging of biomarkers, like alpha synuclein predicting cognitive progression, are proving a better alternative for diagnosing progression [52]. Biomechanical variable quantification, as a response to waist pulls to detect balance control deterioration, is under characterization [53]. Excessive lateral momentum and the increased center of mass and center of pressure (CoP) inclination angle measured in the lateral direction are sensitive measures of loss of equilibrium in elderly patients during gait assessments [33]. Hence, static and dynamic posturography for measuring CoP sway in PI and reactions to an external perturbation in PI have been studied [54,55]. However, these tests are time consuming, expensive, and thus have limited use in a clinical setting.

Development of novel gait and balance analysis techniques have helped in understanding factors that cause instability in PD. For instance, neuroimaging technology brought to light hypoperfusion in the anterior cingulate and primary visual cortex in the PIGD subtype, which is not seen in patients of other PD subtypes [47]. Diffusion tensor imaging (DTI), a noninvasive MRI technique, also differentiates PIGD from controls [38].

Despite the demonstrated accuracy of these tests, scales, and criteria, there have been many delayed diagnoses and misdiagnoses, which is attributed to the subjective nature of these tests. Development of objective tests using potential parameters is needed for accurate, timely diagnosis. Though many pathological factors have been identified, their mechanisms, possible association with each other, and reliability are the major challenges. Once these challenges are met, the measurability of the factors will improve along with the diagnostic adequacy. 

## 5. Factors Contributing to PI in Parkinson’s Disease

**Figure 1:**
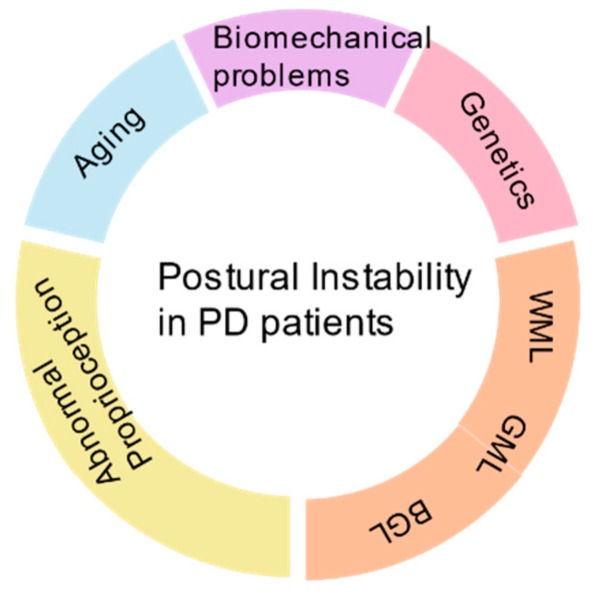
Overview of causative factors of postural instability (PI) in Parkinson’s disease (PD) patients.

Most of the signs and symptoms of PI are nonspecific. Consequently, delays in diagnosis and misdiagnosis arise, which can be prevented by more aggressive and timely investigation. Identification of PI-specific risk factors will help in effective diagnosis and in differentiating the modifiable from nonmodifiable factors to promote prevention. Age, genetic mutations, and race are nonmodifiable, but environmental factors, life style changes, chemical exposures, and stress are modifiable. Measuring the factors might help in effective screening and tracking the disease changes. Some of the identified measurable factors in PI PD patients are fear of fall; biomechanical variables such as center of pressure, center of gravity, and center of mass; age; postural reflexes; defective perception of orientation; impulsivity; and the levels of serum vitamin D. Significant research has been done to understand the correlation of these factors and PI (Figure 1); our article strives to review them.

### 5.1. Age

Levy et al. confirmed that though age is a significant contributing factor for all the motor features of PD, its greatest contribution is to postural instability, more so in the subjects with a higher nondopaminergic sub score [56]. Various age-related changes, such as the sensory changes of the lower limbs, orthostatic hypotension, ability to integrate visual, vestibular, and proprioceptive sensory input, increased latency in muscle responses, and choice of stepping are known to influence the occurrence of PI [2,17,57,58]. All these signify the importance of age as a nonmodifiable risk factor of PI in PD. Moreover, age establishes a new role for neuroprotective agents as a possible therapeutic agent in PD patients [59,60].

Features such as slowness and hesitancy of movements, stooped posture, shuffling gait, and tremor seen in a healthy aged individual are seen in PI PD patients [61,62]. These similarities between the features of age-related stereotypical motor behavior and PD suggest a common etiology between these two motor disorders. However, clinical [62], pathological [63], and biochemical [64] evidence claims that the causes of posture imbalance in healthy older adults and PD patients are not the same [65]. The PD lesions, when diffuse to involve nondopaminergic neurons, result in PI [66]. Since aging factor related changes are not specific to PI in PD (also seen in normal aging), age as an independent measure for PI is unreliable. However, imaging for diffuse neuronal lesions in aged PD patients regularly might specifically help recognize early PI.

### 5.2. Environmental Factors

PD is an interplay between the environment and genes in a susceptible individual. Environmental factors such as living in rural areas, pesticide exposure, coffee consumption, smoking cigarettes, and drinking alcohol are believed to have a role in causing PD [3,67]. Many studies have shown correlation between smoking, alcohol, and caffeine with PD diagnosis [68,69,70,71]. However, only a few have worked to see which subtype of PD has the greatest correlation with the above-mentioned habits. Studies have suggested PI reduction [69,72] with a combination of all the three factors. 

Cigarette smoking stimulates dopamine release, which upregulates nicotinic receptors, and inhibits free radical damage to nigral cells [73]. According to a Norwegian study, there seems to be an inverse correlation between smoking habits and PI subtypes of PD [12].

Caffeine, a nonselective antagonist of adenosine receptors, has been known to decrease the risk of PD [74], with a greater reduction of the PIGD subtype risk when compared to the TD subtype [12,75,76]. Hence, caffeine has also been used in clinical trials to treat imbalance. Escalating the dose of caffeine over a period of six weeks showed a significant improvement of motor symptoms as measured in terms of the UPDRS score [75,76]. Long-term randomized control trials in the future can help tap the therapeutic benefits of caffeine. 

Neuroprotective benefits of moderate alcohol consumption and the occurrence of PD have been a research interest to many scientists [77,78], with a study showing decreased PIGD occurrence with higher alcohol consumption [12].

Since environmental factors have been positive in modifying PI among PD patients, it might prove helpful in treating PI rather than diagnosing. Trials on applicability of the antioxidant properties of alcohol and stimulant effects of nicotine and caffeine might direct future treatment of imbalance. However, it might be useful to screen PD for PI signs if the patient does not have any history of smoking, alcohol, and caffeine consumption.

### 5.3. Genetics

Genetic defects occupy a definite role in the pathogenesis of Parkinson’s disease [79] Mutations known to cause PD are SNCA in aggregating α-synuclein [80], PRKN in impairing proteosomal protein degradation [81], impairment of the oxidative stress response by DJ1 mutation [82], dysfunction of mitochondria by PINK1 mutation [83], cognitive decline due to catechol-O-methyltransferase (COMT) Val/Val mutation [84], and leucine-rich repeat kinase 2 (LRRK2) mutations at the PARK8 locus [20,85,86,87]. 

About 50% to 60% of the LRRK2 mutation carriers are of the PIGD subtype with postural instability as the dominant symptom [88,89,90]. The carriers have a lower tremor, greater postural instability, worse prognosis, and a greater cognitive impairment [91]. These symptoms are independent of age at onset, disease duration, and sex [44,79,89,90]. Thus, LRRK2 mutation screening in PD patients might help shortlist patients at risk for PI. Retrospective clinical trials correlating genetic mutations and PI among PD patients might serve in identifying genetic mutations as an objective factor. 

### 5.4. Nutrition

Nutrients are essential to maintain an individual’s health. Many diseases originate due to altered levels of nutrients in a genetically susceptible individual, and PD is no exception [92]. Epidemiologists and biochemists have revealed the neuroprotective benefits of some food agents such as docosahexaenoic acid (DHA) and vitamin D [92,93,94] in neurodegenerative disorders. Its deficiency might have a role in impairing balance and PD progression [64,95,96,97]. 

Many theories have been postulated relating PI and vitamin D deficiency. Posture is maintained by a complex mechanism involving nervous, motor, and sensory systems, as discussed earlier. Vitamin D receptors are present in muscles and regions like the cerebrum, cerebellum, and spinal cord, which are all involved in complex postural balance mechanisms [98,99]. Hence, it was hypothesized that vitamin D deficiency results in instability. In this regard, researchers have worked to relate vitamin D levels with the severity of symptoms using UPDRS scores and response to tests like external perturbations. Peterson et al. demonstrated a significant, negative correlation between vitamin D levels and PI severity [100]. The Harvard Biomarker study showed a statistically significant correlation between low vitamin D levels and high total motor symptom scores on UPDRS [101,102]. However, correlation does not always signify any causation. 

Several observational and interventional analyses have been conducted to study the effect of supplementation on PI PD patients. Nearly 20% of the neurologically intact community-dwelling elders had their sway reduced after the vitamin D supplementation in its biologically active form, vitamin D3 [100,101,103,104]. In PD subjects, supplementation stabilized motor symptoms and curtailed the increase in the UPDRS score [92,104]. Data from many interventional studies proved that the persistence of low vitamin D levels for a period of time precedes the disease, and the levels do not decrease with disease progression [95,105]. Routine monitoring of the levels of the vitamin D and timely supplementation might prove beneficial in decreasing the imbalance [101,106].

Among all the environmental factors, screening for vitamin D deficiency regularly in PD patients might help curtail postural instability at early stages.

### 5.5. White Matter Lesions

Advancements in brain imaging technology have brought to light the diffuse lesions in white matter occurring in many healthy elderly adults, along with neurodegenerative patients with Alzheimer’s and the Parkinson’s diseases [11,107,108,109,110]. These hypodense lesions on CT scans correlate well with hyperintensity signals of the T2-weighted MRI scans [108,109,110]. They occur due to vascular insults and are termed white matter hyperintensities (WMHs) or periventricular hyperintensities (PVHs) [111,112]. Owing to their location along the long loop tracts, which maintain postural balance, PVHs contribute to balance impairment in the aging population [8,31]. Piccini et al. found that PD with PVH has a faster disease progression with a worse prognosis [107]. PVH is more frequently seen in PD patients than healthy older adults [14]. 

Numerous studies have proposed the role of white matter lesions in the PIGD subtype [8,21,31,107]. Using MRI scans, Lee et al. differentiated PI and TD subtypes based on WMH presence [113]. However, a study relating the WMH burden with the PIGD subtype by Herman et al., using Scheltens’ visual rating scale, showed no significant difference between the mean UPDRS scores [8]. In contrast, Bohnen et al. found a significant association of worsening motor symptoms in PIGD subjects with increasing WMH [14]. This created a knowledge gap in identifying the best method to score PI severity based on WMH. Filling this knowledge gap, a recent clinical trial on 204 PD patients, the Fazeka score and ARWMC scale were used to grade WML severity [114]. These studies serve as evidence for hope in the ability to quantify PI clinically among PD patients.

### 5.6. Gray Matter Atrophy

Neural imaging technology advancements, which have helped in identifying white matter lesions, also helped study gray matter changes. Novel voxel-based morphometry (VBM) analysis strategies paved the way for a deeper understanding of gray matter changes in health and disease. Because of its automatic, time efficient, operator independent, and unbiased whole-brain analyzing nature, VBM has been used widely in neurological disorder diagnosis [115,116]. The same vascular insults that caused the white matter lesions also cause gray matter lesions [117], which extend from the cortex to the subcortical basal ganglia and thalamus gray matter. White matter lesions affect gray matter metabolism and result in atrophy [118]. WMHs decrease the cerebral global blood flow up to 20% [119] and affect glucose metabolism [120]. Such lesions in the substantia nigra cause PD. 

Gray matter helps maintain posture as well as cognition, which are affected primarily in PI PD patients. It was found that gray matter volume decreased in all the regions involved in motor functioning in PD [121]. Though gray matter atrophy had significant correlations with the pull test and instability scores, there was no correlation with the cognitive scores. However, as a part of motor-cognitive interdependence, the decline in cognition explains the difficulty in balance and gait [30,122]. Another study reported that atrophy of gray matter in the pedunculopontine nucleus affects postural adjustments [123]. 

The complex relation between the white and gray matter lesions is largely unexplored and would certainly help in understanding the neurodegenerative process [118]. It is unclear whether the atrophy of gray matter independently, or its combination with WMH, causes instability. The very existence of gray matter atrophy as an independent factor for PI in PD is questionable and needs further research. In this regard, it might be useful to grade higher PI if the patient has both white and gray matter hyperintensities than singly. Scoring PI on pull tests and the ability to maintain adjustments to external perturbations might certainly help grade severity of PI due to gray matter atrophy. This, however, needs extensive future research.

### 5.7. Basal Ganglia Lesions

PI is unresponsive to dopamine replacement therapy. This has led to the realization of new treatment strategies, and one such novel approach is deep brain stimulation (DBS). Currently, DBS surgeries have become an established therapy for motor complications in PD [124]. DBS targets the nuclei of the basal ganglia, which are affected by hypodopaminergy, viz., substantia nigra, striatum, globus pallidus, the subthalamic nuclei, and the ventralis intermedius thalamic nucleus [68,124,125,126,127]. Many studies have reported an improvement of PIGD symptoms with subthalamic nucleus DBS [128,129] in terms of balance and posture [130]. However, a considerable proportion of the patients reported worsening of balance, with an increase in the number of falls [131]. Hence, it is not clear what certainly can measure instability due to basal ganglia lesions—dopamine levels or ability to retain balance to external perturbations. This still needs future research for a clear understanding.

### 5.8. Fear of Fall 

Fear of fall (FoF) refers to the lack of self-confidence to perform balance-related activities of daily living like standing and maintaining certain postures, thus restricting the mobility. Although fear of fall prevails in healthy elderly community dwellers [132,133,134], its prevalence is highest in populations with balance problems such as PD. PD alters balance control strategies to cause PI and falls [126,133,135]. Three factors—postural sway (as measured by the UPDRS), muscle strength, and the confidence (as measured by the ABC score)—contribute to FoF in PI PD subjects [136]. The low balance confidence in PD as measured by the ABC test correlates well with UPDRS motor scores [27,135,136]. Estimating the FoF in PD is necessary, as it helps in preventing future falls because FoF has some grave complications like restricted mobility, decreased functional ability, loss of independence, and social isolation [137]. Considering its complications, reduction in fear of falls is listed as a factor for treating falls in PD [138]. Scoring FoF seems to be one of the best available, objectively gradable strategies to predict PI [139], as FoF has been significantly related to the performance of balance-related motor functions.

### 5.9. Abnormal Proprioception

Balance while standing and walking is a complex high-order form of motor behavior, which depends on several neurological and mental processes [122]. Any abnormality in the input, coordination, or execution of reflexes, reduced peripheral sensation, or oculomotor and labyrinthine functions may contribute to PI [17,29,40]. Reactions to proprioceptive disturbances are abnormal in PI PD patients [55]. They compensate this loss with visual [140] and auditory [141] cues. The scaling and habituation of erroneous proprioceptive information are affected in advanced stages of PD, resulting in PI [41], and the visual input no longer can compensate, thus resulting in the loss of stability. They develop abnormal strategies for stimuli such as selecting inappropriate and poorly coordinated responses, which are fixed irrespective of the functional demands [17], lack anticipation of the reflexes [61], and demonstrate poor control of motor impulses [42]. 

Hence, proprioceptive input analyses affected in PD patients causes PI. Research has made it clear that inputs in the form of auditory and visual cues are critical in compensating the proprioceptive loss in patients. These cues can be used in the treatment of instability in patients [140,141]. However, to confirm its curative role, longitudinal clinical trials are needed. Proprioceptive input analysis, a complex analysis involving input from different sensory centers and the response of the patient to known and unknown sensory cues, when staged and graded critically, can effectively diagnose the stage of PI in PD patients.

### 5.10. Biomechanical Variables

Early detection of imbalance in PD patients before the appearance of clinical symptoms is very crucial to prevent falls. Identification of certain biomarkers, chemical and mechanical, by laboratory experiments to sense the instability and establish fall risk has begun.

Even before the clinical diagnosis of PI, the movement preparation phase is most affected by PD because of the loss of automatic selection and execution of motor plans attributed to the basal ganglia lesion [57,142]. Center of pressure (CoP), Center of gravity (CoG), and gait analysis variables like the step length, velocity, and choice of stepping are some of the potential biomechanical parameters that can help us learn the effect of PD on stability. CoP measurements by many research groups have proven its alterations in PD patients [53,143]. Moderate to severe PD alters the step length while responding to balance perturbations [144]. Gait initiation requires simultaneous forward movement and balance control. During a normal walk, the CoG moves out of the support base and collapses, and the body loses balance when the swing leg is lifted. For movement continuation, further collapse in the CoG should be broken with the help of antigravity muscles [145]. This brake in CoG collapse is impaired in PI PD patients, resulting in imbalance and falls [133,146]. Similarly, in a clinical study, poor trunk movements differentiated a faller from a nonfaller [147]. All these features are easy to measure, and their validity can be proved effectively. 

CoG, CoP, and trunk movement abnormality measurements clearly are measurable biomechanical variables that can help diagnose PI.

## 6. Conclusions

PI, the most disabling feature of PD, has emerged as the greatest challenge in PD management. The only way to meet this challenge is by careful screening and timely diagnosis, along with effective intervention, to prevent any complication of falls. PI management will prove effective only when the etiology is specifically identified and properly treated. Identifying the specific etiology is difficult because of the heterogeneous nature of the disorder along with the lack of knowledge about the pathogenesis of the disease. From research conducted so far, PD patients could have PI due to multiple reasons. Hence, there is a need to search for specific causes personalized to patients. Significant research has been done to identify the pathogenic factors of PI. Despite the identification of many factors, their validity is questionable. Deficits like subjective nature of the diagnostic procedures, validity of etiologic factors, along with difficulty in measuring the instability are the issues to be resolved. 

Instability measurements have been, so far, subjective with inter clinician variability. Hence, there is a need for an objective type of diagnosis. Objective testing tools have been in use only in research labs because of their laborious, time consuming, and expensive nature. Our review on currently identified factors suggests: (a) CoP, CoG, and trunk movements are measurable biomechanical markers of PI; (b) vitamin D levels and LRRK2 mutations are screening measures that can be routinely done to identify PD patients at risk for PI; (c) WMH, GM atrophy, and basal ganglion lesions are the pathological factors to be identified using imaging modalities, which could be scored based on tests like the pull test; (d) periodic monitoring of visual and auditory capabilities of a PD patient; and (e) the proprioceptive capacity maintains balance and FoF levels while responding to external perturbations. Developing a PI diagnostic chart including all the factors discussed in our review might serve as a useful tool. The diagnostic chart will include three levels: screening, objective diagnosis, and pathology identification. If a patient scores positive for one level, the next level of diagnosis becomes mandatory. We speculate that early identification of altered criteria in either of the diagnostic levels will help curtail PI severity in PD patients. Validation and quantification to correlate the severity of these factors and PI could be a subject of future research. Overall, it is worth conducting clinical trials to correlate objective factors with PI pathology and symptoms. Developing devices that are cost effective in diagnosing biomechanical variables seems to be the future of effective diagnosis of PI and other movement disorders as well. It is critical to diagnose movement disorders early on to prevent falls and fall-associated emotional and economic burden.
